# The Antiviral Roles of Hydrogen Sulfide by Blocking the Interaction between SARS-CoV-2 and Its Potential Cell Surface Receptors

**DOI:** 10.1155/2021/7866992

**Published:** 2021-09-03

**Authors:** Jing Dai, Xu Teng, Sheng Jin, Yuming Wu

**Affiliations:** ^1^Department of Clinical Diagnostics, Hebei Medical University, Hebei 050017, China; ^2^Department of Physiology, Hebei Medical University, Hebei 050017, China; ^3^Hebei Collaborative Innovation Center for Cardio-Cerebrovascular Disease, Hebei 050017, China; ^4^Key Laboratory of Vascular Medicine of Hebei Province, Hebei 050017, China

## Abstract

The ongoing coronavirus disease 2019 (COVID-19) pandemic caused by severe acute respiratory syndrome coronavirus 2 (SARS-CoV-2) is posing a great threat to the global economy and public health security. Together with the acknowledged angiotensin-converting enzyme 2, glucose-regulated protein 78, transferrin receptor, AXL, kidney injury molecule-1, and neuropilin 1 are also identified as potential receptors to mediate SARS-CoV-2 infection. Therefore, how to inhibit or delay the binding of SARS-CoV-2 with the abovementioned receptors is a key step for the prevention and treatment of COVID-19. As the third gasotransmitter, hydrogen sulfide (H_2_S) plays an important role in many physiological and pathophysiological processes. Recently, survivors were reported to have significantly higher H_2_S levels in COVID-19 patients, and mortality was significantly greater among patients with decreased H_2_S levels. Considering that the beneficial role of H_2_S against COVID-19 and COVID-19-induced comorbidities and multiorgan damage has been well-examined and reported in some excellent reviews, this review will discuss the recent findings on the potential receptors of SARS-CoV-2 and how H_2_S modulates the above receptors, in turn blocking SARS-CoV-2 entry into host cells.

## 1. Introduction

The ongoing coronavirus disease 2019 (COVID-19) pandemic has now spread worldwide to more than 200 countries/regions and has caused over 180 million infections, and over 4 million deaths globally (as of 10 July 2021), which continues to rise rapidly. It is posing a great threat to the global economy and public health security. The current pandemic is caused by the novel severe acute respiratory syndrome coronavirus 2 (SARS-CoV-2), a lipid-enveloped positive-sense RNA virus belonging to the *β*-coronavirus genus. Similar to other *β*-coronaviruses, spike (S) protein mediates attachment and membrane fusion of virus particles with target cells in SARS-CoV-2 infection [1]. The S protein is a typical type I fusion protein and is composed of two functional subunits: S1, which contains the receptor binding domain (RBD) to mediate receptor binding, and S2, which contains the transmembrane domain to mediate virus-cell fusion [2]. Together with the acknowledged angiotensin-converting enzyme 2 (ACE2), [3] glucose-regulated protein 78 (GRP78), [4] transferrin receptor (TFR), [5] AXL, [6] kidney injury molecule-1 (KIM-1) [7], and neuropilin 1 (NRP1) [8] are identified as additional potential receptors to mediate SARS-CoV-2 infection. The first step of SARS-CoV-2 infection in humans is the binding of RBD in the S1 subunit to the host's cell surface receptors, which plays a decisive role in the invasion and spread of viruses, and, in turn, affects the clinical symptoms of patients. Therefore, how to inhibit or delay the binding of RBD with the abovementioned receptors is a key step for the prevention and treatment of COVID-19.

For a long time, hydrogen sulfide (H_2_S) was known as a poisonous gas to life and the environment. However, since the pioneering work by Abe and Kimura in which it was reported as a neuromodulator, H_2_S has been recognized as the third gasotransmitter akin to nitric oxide and carbon monoxide [9]. In biologic systems, H_2_S is endogenously synthesized by three enzymes, namely, cystathionine *γ*-lyase (CSE), cystathionine *β*-synthase (CBS), and 3-mercaptopyruvate sulfurtransferase (3-MST) and elicits its effect through four distinct pathways: (1) scavenging reactive oxygen species (ROS); (2) posttranslational modification, termed S-sulfhydration or persulfidation, on protein cysteine residues; (3) binding of metalloprotein centers; and (4) interaction with inter- or intramolecular disulfide bonds. [10–12] It is becoming increasingly clear that H_2_S plays an important role in many physiological processes, and disturbances of the endogenous H_2_S production are associated with the onset of several diseases, such as hypertension, diabetes, cancer, and viral infection. [13–15] Recently, Renieris et al. found that survivors had significantly higher H_2_S levels in COVID-19 patients and mortality was significantly greater among patients with a decrease of H_2_S levels. [16] Combined application of N-acetylcysteine, a potential H_2_S-releasing donor, improved the symptoms in COVID-19 patients [17]. Furthermore, a beneficial role of H_2_S against COVID-19 and COVID-19-induced comorbidities and multiorgan damage had been well examined and reported in some excellent reviews [18, 19]. Here, this review will discuss the recent findings on the potential receptors of SARS-CoV-2 and how H_2_S modulates the abovementioned receptors, in turn blocking SARS-CoV-2 entry into host cells.

## 2. Organ Damage of the SARS-CoV-2 and the Protective Effect of H_2_S

Similar to other coronaviruses, direct organ damage will be induced by the SARS-CoV-2 replication once it has invaded the host cell. [20–22] Then, it also can induce organ damage indirectly by the systemic inflammatory response (also called as cytokine storm) [23], endothelial dysfunction, [24] hypoxia, [25] and sympathetic overactivation. [26] Although high H_2_S concentration is cytotoxic by inhibition of mitochondrial respiration, the physiological concentration of H_2_S has been reported to protect multiple organs from injury by its broad spectra of bioactivities, including antiviral, alleviation of inflammation, restoration of endothelial function, inhibition of the hypoxia or ischemia injury, and normalization of sympathetic activities. [19, 27–29] Firstly, accumulated evidence has demonstrated that H_2_S significantly decreased viral replication and improved lung functions in mice, while blockage of CSE activity or knockout of CSE expression increased viral replication and enhanced lung damage. [30] H_2_S also upregulated ACE2 expression to reduce organ damages that were exacerbated by Ang II accumulation after ACE2 internalizaion [31]. Secondly, after being released, viral RNA, as a pathogen-associated molecular pattern, was recognized by a variety of pattern recognition receptors (PRRs) including Toll-like receptors (TLRs) in an immune cell. Then, large amounts of proinflammatory cytokines and chemokines were secreted in an unrestrained way causing a cytokine storm and serious organ damage [32]. H_2_S was found to reduce the expression of TLRs to prevent TLR-mediated inflammatory response. [33] H_2_S was also found to inhibit the secretion of virus-induced chemokines and cytokines by inhibiting the activation and nuclear translocation of NF-*κ*B and then reducing the transcription of proinflammatory genes [34]. Thirdly, Varga et al. verified that endothelial cells were directly infected by SARS-CoV-2 and caused diffuse endothelial inflammation, which induced endothelial dysfunction, thereby worsening organ damage. [35] H_2_S was reported in various studies to ameliorate endothelial dysfunction in cardiovascular disorders such as hypertension, atherosclerosis, and metabolic syndrome, [13, 27] which would be beneficial for COVID-19 treatment. Fourthly, in addition to virus-related lung damage, macro- and microvascular thrombosis induced by inflammation and endothelial dysfunction could cause tissue hypoxia and aggravate organ damage [36]. It was worthy of mentioning that H_2_S has been identified as an excitatory mediator of hypoxic sensing in the carotid bodies to elicit its protective roles under hypoxic conditions. [37] H_2_S was also found to attenuate ferric chloride-induced arterial thrombosis and enhance the blood flow. [38] In addition, it promoted angiogenesis and increased capillary density to limit damages in the ischemic tissues. [39] Finally, it was indicated that patients with preexisting cardiovascular diseases, including hypertension, diabetes mellitus, and ischemic heart disease, which were characterized by increased sympathetic activity, seem to have a higher risk of morbidity and mortality in COVID-19. Conversely, COVID-19 also increased sympathetic overactivation inducing organ damage. [40] The vicious circle between COVID-19 and sympathetic overactivation might exacerbate the organ damage and comorbidities. However, H_2_S could break this vicious circle by inhibiting sympathetic activation in the significant central sympathetic sites [41, 42].

## 3. The Potential Receptors of SARS-CoV-2 and H_2_S

The virus-induced organ damage as described above is initiated by the binding of S protein with the host's cell surface receptors. Here, the important entry receptors including ACE2, GRP78, TFR, AXL, KIM-1, and NRP1 have been outlined, and the possible mechanism of H_2_S in blocking SARS-CoV-2 entry has been discussed (Table 1).

## 4. ACE2 and H_2_S

ACE2, a type I integral membrane glycoprotein composed of a single extracellular N-terminal domain containing the active catalytic site domain, a C-terminal membrane anchor, and a HEXXH zinc-binding domain, is widely expressed in a variety of tissues and cell types, including those in the lungs, heart, kidneys, gut, and brain [86]. Known as a typical zinc metallopeptidase, ACE2 counterregulates the renin-angiotensin-aldosterone system (RAAS) by converting Ang II to Ang 1-7 or Ang I to Ang 1-9, thus maintaining blood pressure homeostasis and fluid and salt balance. It also functions as an amino acid transporter or a functional receptor for MERS-CoV and SARS-CoV. [87] Like in SARS-CoV infections, ACE2 serves as a major entry receptor for SARS-CoV-2 in humans by binding to its S protein. [88] ACE2 has a 10- to 20-fold higher affinity for SARS-CoV-2 S than for SARS-CoV, which results in a higher SARS-CoV-2 infection efficiency. [89] Recent evidence in the literature indicated that intra- and intermolecular disulfides in both ACE2 and SARS-CoV-2 S protein had an important role for the binding process, which was regulated by the thiol-disulfide balance of the extracellular environment. [43, 44] Using molecular dynamics simulations revealed that the reduction of all disulfides into the sulfydryl groups completely impaired the binding of the SARS-CoV-2 S protein to ACE2. When the disulfides of only ACE2 were reduced to sulfydryl groups, the binding became weaker, while the reduction of disulfides of the SARS-CoV-2 S protein had a comparatively less effect [45]. Recently, several reducing agents including N-acetylcysteine amide and L-ascorbic acid were reported to inhibit viral entry by the disruption of disulfides [46]. As a weak reducing agent, H_2_S activated vascular endothelial growth factor receptor 2 (VEGFR2) to promote angiogenesis by breaking the Cys1045-Cys1024 disulfide bond within the receptor [47]. H_2_S also targeted the Cys320/Cys529 motif and broke the disulfide bonds in Kv4.2 to inhibit I_to_ potassium channels in cardiomyocytes and regularize fatal arrhythmia in myocardial infarction [48]. Moreover, H_2_S was used to break mucin disulfide bonds, making the mucus less viscous and easier to be expelled by the respiratory ciliary apparatus, facilitating the elimination of potentially harmful viruses or extraneous particle [49]. Thus, H_2_S is hypothesized to exhibit antiviral activity by interfering with the combination of ACE2 and SARS-CoV-2.

## 5. CS-GRP78 and H_2_S

GRP78, also known as immunoglobulin heavy-chain-binding protein (BiP) or heat shock protein A5 (HSPA5), is a well-characterized endoplasmic reticulum (ER) chaperone protein whose function is to translocate nascent polypeptides across the ER membrane and facilitates the correct folding and assembly of proteins in normal cells. When misfolded proteins accumulate in the ER following ER stress, GRP78 is upregulated and plays a pivotal role in the unfolded protein response (UPR) by binding to misfolded proteins initiating the refolding or degradation mechanisms [90]. Conversely, under the ER stress, overexpressed GRP78 can escape the ER retention and translocate to the cell surface, termed cell surface CS-GRP78, where it functions as a multifunctional receptor to regulate cellular signaling, proliferation, migration, invasion, apoptosis, inflammation, and immunity [91]. CS-GRP78 is also reported to play a critical role in viral and fungal infections. Viruses including Coxsackie virus, Zika virus, dengue virus, and Borna disease virus recognize CS-GRP78 for entry or invasion into the host cells. [92] CS-GRP78 was reported to facilitate MERS-CoV entry into permissive cells by augmenting virus attachment in the presence of DPP4 [93]. Recently, a molecular dynamics study combined with molecular docking revealed the existence of H-bonds or hydrophobic contacts between GRP78 and C480-C488 of SARS-CoV-2 S protein [4, 94], which might be related to viral infection. A better binding was also found between GRP78 and the new UK variant of SARS-CoV-2. [95] In addition, COVID-19 patients had higher gene expression and serum concentrations of GRP78 [96]. Considering that virus invasion was associated with elevated levels of CS-GRP78 expression, inhibiting overexpressed GRP78 would be a promising strategy to reduce virus infection. H_2_S has been reported to downregulate the expressions of ER stress-related proteins, including GRP78, in multiple diseases by different pathways. Our study found that the ER stress markers, including GRP78, CHOP, and active caspase-12 levels, were significantly elevated in the calcified rat aorta and H_2_S alleviated vascular calcification by inhibiting ERS through the Akt signaling pathway activation [50]. In uranium-treated kidney cells, H_2_S downregulated the expressions of GRP78 and CHOP and attenuated ER stress via 20S proteasome involved in Akt/GSK-3*β*/Fyn-Nrf2 signaling axis. [51] In hyperhomocysteinemia-induced cardiomyocyte injury, H_2_S supplementation decreased the expressions of ER stress-associated proteins, including GRP78, while the inhibition of endogenous H_2_S production further increased the expressions of those proteins [52]. H_2_S was also reported to inhibit cigarette smoke-induced overexpression of ER stress-associated proteins in bronchial epithelial cells [53]. Therefore, H_2_S may block the SARS-CoV-2 from entering the host cells by inhibiting the ES stress and reducing the expression of CS-GRP78.

## 6. TFR and H_2_S

TFR is a membrane receptor playing a critical role in the maintenance of body iron homeostasis. The TFRs have two subtypes: TFR1 and TFR2. TFR1 is ubiquitously expressed at different levels on normal cells and serves as a gatekeeper regulating the cellular uptake of iron from transferrin, while TFR2 is specially expressed in hepatocytes and serves as an iron sensor [97]. TFR1 has attracted more attention than TFR2 by having diverse functions. TFR1, also known as cluster of differentiation 71 (CD71), is a homodimeric type II transmembrane glycoprotein involved in the cellular iron uptake through a constitutive clathrin-dependent endocytosis mechanism. It is expressed at low levels in most normal cells and at greater levels in rapidly proliferating cells and energy-requiring cells owing to the increased iron requirements. So, it may play additional roles in cell growth and proliferation [98]. Given that it is a ubiquitously and abundantly expressed cell surface membrane protein, TFR1 is a vulnerable target for pathogens to initiate host cell infection. It has been documented that multiple viruses, including New World hemorrhagic arenaviruses, hepatitis C virus, and human adenoviruses, recognize and bind with the apical domain of TFR1 to enter cells without interfering with iron delivery. [99] In this way, the viruses infect rapidly proliferating and iron-acquiring cells, which can facilitate their replication. Furthermore, endocytosed TRF1 recycles back to the cell surface in a constitutive manner but is not downregulated by viruses' infection, which may cause the superinfection. [100] Recently, it was reported that TFR1 directly interacted with the S protein of SARS-CoV-2 to mediate virus entry, while it was blocked by interfering TFR-spike interaction. Furthermore, anti-TFR antibody showed the promising antiviral effects in mouse model. [5] Considering that it is another receptor for SARS-CoV-2 entry, downregulating the expression of TFR1 or preventing the translocation of TFR1 to plasma membrane may be effective strategies to prevent virus invasion. Various molecular mechanisms are involved in the regulation of its expression at both the transcriptional and posttranscriptional levels. At the transcriptional level, TFR1 gene transcription has been shown to be stimulated by the transcription factor c-Myc. [54] However, the impact of H_2_S on the c-Myc remains controversial. Zhang et al. reported that exogenous H_2_S activated the ERK1/2/c-Myc pathways and restored postconditioning-mediated cardioprotection in the aged cardiomyocytes [101]. Contrastingly, Song et al. reported that H_2_S donor inhibited the cell proliferation by downregulating the expression of proliferation-related proteins including c-Myc. [55] Moreover, diallyl disulfide, a potential H_2_S donor, decreased telomerase activity in U937 cells by reduced binding of c-Myc to their respective binding sites on the promoter. [56] At the posttranscriptional level, TFR1 expression is finely regulated in an intracellular iron-dependent manner by the iron-responsive element/iron-regulated protein (IRE/IRP) system. [57] In case of cellular iron deficiency, the two IRPs (IRP1 and IRP2) bind to the multiple IRE motifs by the -SH residues in the 3′ untranslated region of TFR1 mRNA and inhibit their degradation by a steric hindrance mechanism, thus increasing TFR1 protein expression. Conversely, in the presence of excess iron, IRP1 becomes an aconitase with the binding of a 4Fe-4S cluster, while IRP2 is degraded after ubiquitination, leading to the disappearance of IRE binding activity and degradation of TFR1 mRNA. Reactive oxygen species (ROS), including superoxide anion and hydrogen peroxide, was found to promote the loss of the 4Fe-4S cluster and enhance the IRE binding activity of IRP1, resulting in TFR1 translation [58]. Since it has long been assumed to be an antioxidant, H_2_S may inhibit the IRP binding activity and downregulated TFR1 protein by scavenging ROS. H_2_S was also reported to regulate the bioactivities of multiple proteins via S-sulfhydration of cysteine residues, [59] so H_2_S might S-sulfhydrate cysteine residues of IRP1 to prevent its IRE binding activity, thus downregulating TFR1 protein. In addition, the Na^+^/H^+^ exchanger enhanced TFR1 translocation to the membrane of microvascular endothelial cells at the blood-brain barrier, [60] which might be inhibited by H_2_S [102].

## 7. AXL and H_2_S

AXL, also known as UFO, ARK, Tyro7, or JTK11, belongs to the tumor-associated macrophage (TYRO3, AXL, and MERTK) family receptor tyrosine kinases (RTKs). After binding with its ligand, growth arrest-specific protein 6 (GAS6), it leads to the activation of several downstream signaling pathways, including the Ras/Raf/MEK/ERK cascade and PI3K/Akt signaling pathways, and transduces signals from the extracellular matrix into the cytoplasm. [103] AXL has been originally detected as an unidentified transforming gene in chronic myeloid leukemia. Since then, AXL is found to be overexpressed in many types of cancer and is associated with therapy resistance, adverse prognosis, and worse outcome [104]. Under normal physiologic conditions, AXL is ubiquitously expressed in a wide variety of organs and cells originating from hematopoietic, epithelial, and mesenchymal sources and regulates many important physiological processes, including taming inflammation, clearing apoptotic cells, maintaining vascular integrity, and regulating cell survival, proliferation, and differentiation. Moreover, AXL has been found to be a candidate entry receptor for West Nile, Ebola, and Zika viral infections and its specific inhibitors reduced viral infectivity [105]. Most recently, Wang et al. found that AXL specifically interacted with the N-terminal domain of the spike glycoprotein in SARS-CoV-2, which colocalized mainly to the cell membrane, and it was a novel entry receptor for SARS-CoV-2 which played an important role in promoting viral infection to the human respiratory system. [6] In line with it, gilteritinib, an AXL inhibitor for acute myeloid leukemia treatment, was recently demonstrated to possess antiviral efficacy against SARS-CoV-2 infection in Vero E6 cells [106]. After virus infection, the TLR-mediated immune network is stimulated by viral particles, and then, the consequent type I interferon (IFN) antiviral response upregulates AXL expression, [107] which further promotes virus infectivity. Huang et al. reported that H_2_S downregulated TLR4, inhibited its downstream NLRP3 inflammasome activation, and alleviated high glucose-induced cardiac injury [108]. H_2_S was also able to ameliorate LPS-induced inflammation through TLR4/NF-*κ*B signaling pathway inhibition [109]. In addition, polysulfide donors were reported to protect the mice from lethal endotoxin shock by inhibiting TLR signaling. [110] Several transcription factors, including specificity protein 1 (Sp1) [61] and hypoxia-inducible factor 1*α* (HIF-1*α*), [63] have been shown to directly upregulate AXL expression at transcriptional levels. H_2_S-mediated S-sulfhydration of the Sp1 has been shown to decrease its binding activity to the gene promoter region, thus preventing myocardial hypertrophy [62]. H_2_S also suppressed HIF-1*α* translation or activation under hypoxia [64, 65]. Conversely, reduced H_2_S levels increased the levels of HIF-1*α* via increased ROI levels in infected CSE KO macrophages [111]. Histone acetylation can also affect AXL transcript levels. Reduced histone acetylation of the AXL promoter led to the upregulation of AXL expression that correlated with therapy resistance and adverse prognosis in some types of cancers [66]. AOAA, the inhibitor of endogenous H_2_S production, has been reported to reduce histone acetylation, and H_2_S donor increased H3 and H4 acetylation in LPS-treated cell [67]. H_2_S also suppressed the endothelial dysfunction and prevented the occurrence of hypertension by inhibiting HDAC6 expression that removes acetyl groups from lysine residues of histone to reverse histone acetylation [68]. In addition, AXL mRNA expression is inhibited by miR-34a which has identified target sequences in the AXL 3′ untranslated region. [112] miR-34a expression was found to upregulate diallyl disulfide-treated MDA-MB-231 cells [113].

## 8. KIM-1 and H_2_S

KIM-1, also known as TIM-1, is a single-pass type I cell membrane glycoprotein with an extracellular six-cysteine immunoglobulin-like (Ig V) domain topping a domain characteristic of mucin-like O-glycosylated proteins. It is virtually undetectable in normal kidney tissues, but its expression is dramatically upregulated in the apical membrane of the proximal tubule to reduce the innate immune response and regulate the regeneration and repair of the damaged epithelial cells after acute ischemic or toxic kidney injury. However, prolonged KIM-1 expression may be maladaptive and may lead to interstitial inflammation and fibrosis in chronic kidney disease. Therefore, it is recognized as a robust and reliable biomarker for early diagnosis, prognosis, and monitoring of therapeutic effects in various kidney diseases [114]. Moreover, KIM-1 is also identified as a hepatitis A virus cell receptor 1 (HAVCR-1) that is expressed by on the surface of different epithelial cells and facilitates cellular entry of several viruses, including Ebola virus, dengue virus, West Nile virus, and hepatitis A virus, via the IgV domain [115]. A recent report suggested that KIM-1 was not only a biomarker for COVID-19-associated acute kidney injury (AKI) [116] but also a potential receptor for SARS-CoV-2. [7] SARS-CoV-2 was reported to directly infect the renal tubules by ACE2 and induced AKI, which is one of the most prevalent complications among hospitalized COVID-19 patients [117]. After upregulated expression induced by AKI, KIM-1 could directly bind to SARS-CoV-2 S protein which was inhibited both by anti-KIM-1 antibodies and TW-37, an inhibitor of KIM-1 [118]. Another study suggested that SARS-CoV-2 RBD bind with KIM-1 and ACE2 via two distinct pockets, implicating that KIM-1 and ACE2 may synergistically mediate the invasion of SARS-CoV-2 in kidney cells [119]. The above “vicious cycle” exacerbates SARS-CoV-2 infection and KIM-1 may offer a new therapeutic target that can minimize injuries due to SARS-CoV-2. It was reported that H_2_S treatment downregulated KIM-1 expression in hyperglycemic condition by inhibiting Ca^2+^-induced mitochondrial permeability transition pore opening [120]. Dopamine decreased KIM-1 levels and preserved renal integrity during deep hypothermia and rewarming likely by maintaining the expression of renal H_2_S-producing enzymes and serum H_2_S. [121] A previous report had shown that nuclear signal transducer and activator of transcription 3 (STAT3) could bind to the KIM-1 promoter and increased its mRNA and protein levels. [69] In our study, PPG, the inhibitor of endogenous H_2_S production, increased phosphorylation of STAT3 and aggravated vascular remodeling, while NaHS decreased phosphorylation of STAT3 and improved vascular remodeling [70]. The AMPK pathway might mediate the inhibition of STAT3 phosphorylation by H_2_S during inflammation [71]. Recently, polysulfides were also reported to attenuate diabetic renal lesions via the inactivation of STAT3 phosphorylation/acetylation through S-sulfhydrating SIRT1. [72] In addition, the increased KIM-1 expression was also mediated by the ROS or ERK1/2 pathway, [73, 74] whereas H_2_S not only attenuated ROS production but also abolished ERK1/2 activation, which possibly decreased KIM-1 expression. [75]

## 9. NRP1 and H_2_S

Neuropilins (NRPs) are highly conserved single-pass transmembrane glycoproteins that are expressed by a wide variety of cell types, including neurons, blood vessels, immune cells, and multiple tumor cells in mammals. To date, two homologous NRP isoforms have been identified, namely, NRP1 and NRP2, which share 44% sequence homology and have a similar domain structure. The NRPs are composed of a large extracellular domain, a transmembrane domain, and a short cytoplasmic domain that lacks enzymatic activity. Despite being devoid of an intracellular kinase domain, NRPs act predominantly as a multifunctional coreceptor to bind with various ligands including class 3 semaphorins (SEMA3s), vascular endothelial growth factor, fibroblast growth factor, and transforming growth factor-*β*1 (TGF-*β*1) by their well-structured extracellular part. As such, NRPs mediate a wide range of signaling pathways and play critical roles in the physiological and pathological processes, including nervous and vascular development, immune response, and tumor progression. [122] Moreover, NRPs have been shown to mediate cellular entry and infectivity of viruses such as Epstein-Barr virus (EBV), human T cell lymphotropic virus-1 (HTLV-1), and murine cytomegalovirus (MCMV) [123–125]. Recent literature has established NRP1 as a coreceptor that facilitated SARS-CoV-2 cell entry and infectivity, and NRP1 mRNA expression was elevated in SARS-CoV-2-infected cells, but not in uninfected cells from severe COVID-19 patients [126]. Studies based on X-ray crystallography and biochemical approaches also showed that the SARS-CoV-2 S proteins directly bind with extracellular domain of NRP1 by electrostatic attraction and infected human cells. [8] So NRP1 could be an ideal therapeutic target against SARS-CoV-2 infections. Although it lacks a direct study, H_2_S may indirectly regulate NRP1 expression by affecting its transcription factors or some cytokines. It had been demonstrated that NRP1 was the downstream target of transcription factor Sp1 or HIF-1*α* [76, 77]. However, as mentioned above, H_2_S inhibited the downstream protein expression by regulating these two transcription factors [62, 64, 65]. Cytokines, such as TNF-*α* and TGF-*β*, were reported to induce NRP1 mRNA and protein expressions, [78, 79] while, as indicated by the plethora of evidence, H_2_S downregulated TNF-*α* and TGF-*β* expressions in a variety of pathological conditions [80, 81]. In addition, NRP1 was upregulated by Wnt/*β*-catenin signaling and sonic hedgehog (SHH)/GLI1 signaling in mammary development and tumorigenesis [82, 83]. However, diallyl trisulfide, a H_2_S donor, was found to inhibit breast cancer stem cells via suppression of the Wnt/*β*-catenin pathway, and sulforaphane, another H_2_S donor, significantly inhibited the SHH/GLI1 pathway and its downstream target gene expression to regulate self-renewal of pancreatic cancer stem cells [84, 85].

## 10. Conclusion

This review summarizes the potential receptors for entry of SARS-CoV-2, including GRP78, TFR, AXL, KIM-1, and NRP1, in addition to ACE2. Meanwhile, the potential mechanism by which H_2_S regulates the abovementioned receptors to block the binding of SARS-CoV-2 has been discussed. Although inorganic sulfide salts (NaHS and Na_2_S) have been the most widely employed in biological and preclinical studies, none of them are unlikely to be a suitable clinical option for a number of reasons, including poor water solubility, fast and uncontrollable release, and unpleasant odor. Given that it is not trivial to synthesize a clinically suitable H_2_S donor in a short time, three types of potential H_2_S donors or drugs should be considered to block viral entry: (1) natural H_2_S donors (e.g., garlic and onions) [127, 128] or dietary micronutrients (e.g., L-cysteine and taurine) [129, 130], (2) H_2_S-donating derivatives of clinically used drugs that link various H_2_S donating groups to clinically used drugs (e.g., ATB-346 [131] and GIC-1001 [132] which has completed phase 2 clinical trial), and (3) several clinically used drugs that have been verified to increase H_2_S levels (e.g., *α*-lipoic acid, [133] sodium thiosulfate, [134] zofenoprilat, [135] and N-acetylcysteine [136]). Sodium thiosulfate has been proposed as an inhalation therapy for COVID-19, [137] and it was confirmed that the combined application of N-acetylcysteine improved the symptoms in COVID-19 patient. [17] However, the abovementioned is just a stopgap; developing new clinically suitable H_2_S donor drugs is necessary, and the clinical application of H_2_S-targeted therapeutics to fight against diseases that are not limited to COVID-19 should advance.

## Figures and Tables

**Table 1:** Potential host receptors and possible mechanism of H_2_S in blocking SARS-CoV-2 entry.

Potential receptors	Possible regulatory mechanism of H_2_S	Key references
ACE2	Interaction with intramolecular disulfide bonds	[43–49]
GRP78	Downregulation of its expression by inhibiting the related signal pathways	[50–53]
TFR	Downregulation of its expression at transcriptional levels	[54–56]
	Downregulation of its expression at posttranscriptional levels	[57–60]
AXL	Downregulation of its expression by S-sulfhydrating its transcription factor	[61, 62]
	Downregulation of its expression by inhibiting its transcription factor translocation	[63–65]
	Downregulation of its expression by histone acetylation of its promoter	[66–68]
KIM-1	Downregulation of its expression by reducing the phosphorylation of its transcription factor	[69–72]
	Downregulation of its expression by reducing ROS or ERK1/2 pathway	[73–75]
NRP1	Downregulation of its expression by S-sulfhydrating its transcription factor	[62, 76]
	Downregulation of its expression by inhibiting its transcription factor translocation	[64, 65, 77]
	Inhibition of cytokines	[78–81]
	Downregulation of its expression by inhibiting the related signal pathways	[82–85]
